# Iterative development of Vegethon: a theory-based mobile app intervention to increase vegetable consumption

**DOI:** 10.1186/s12966-016-0400-z

**Published:** 2016-08-08

**Authors:** Sarah A. Mummah, Abby C. King, Christopher D. Gardner, Stephen Sutton

**Affiliations:** 1 Behavioural Science Group, Institute of Public Health, University of Cambridge, Cambridge, UK; 2 Stanford Prevention Research Center, Department of Medicine, Stanford University School of Medicine, Stanford, CA USA

**Keywords:** mHealth, Smartphone, Mobile, Diet, Vegetables, Process motivators, Persuasive design, Behavior design, User-centered design, Design thinking

## Abstract

**Background:**

Mobile technology may serve as a cost-effective and scalable tool for delivering behavioral nutrition interventions. This research sought to iteratively develop a theory-driven mobile app, *Vegethon*, to increase vegetable consumption.

**Methods:**

Development of Vegethon followed phases outlined by the IDEAS framework: 1) empathize with users (qualitative interviews, *n* = 18); 2) specify target behavior; 3) ground in behavioral theory; 4) ideate implementation strategies; 5) prototype potential products; 6) gather user feedback (qualitative interviews, *n* = 14; questionnaire, *n* = 41); 7) build minimum viable product; and 8) pilot potential efficacy and usability (pilot RCT, *n* = 17). Findings from each phase informed subsequent phases. The target population that informed intervention development was 18–50 years of age, had BMIs of 28–40 kg/m^2^, and lived in the geographical area surrounding Stanford University. A full description of the final version of Vegethon is included in the paper.

**Results:**

Qualitative findings that shaped initial intervention conception were: participants’ interests in accountability without judgment; their desire for simple and efficient dietary self-monitoring; and the importance of planning meals in advance. Qualitative findings identified during intervention refinement were the need for a focus on vegetable self-monitoring; inclusion of vegetable challenges; simplification of features; advice and inspiration for eating vegetables; reminder notifications; and peer comparison. Pilot RCT findings suggested the initial efficacy, acceptance, and feasibility of the intervention. The final version of Vegethon enabled easy self-monitoring of vegetable consumption and included a range of features designed to engage the user (e.g., surprise challenges; leaderboard; weekly reports). Vegethon was coded for its inclusion of 18 behavior change techniques (BCTs) (e.g., goal setting; feedback; social comparison; prompts/cues; framing/reframing; identity).

**Conclusions:**

Vegethon is a theory-based, user-informed mobile intervention that was systematically developed using the IDEAS framework. Vegethon targets increased vegetable consumption among overweight adults and is currently being evaluated in a randomized controlled efficacy trial.

**Trial registration:**

Clinical Trials.gov: NCT01826591

## Background

Poor diet is among the leading causes of death in the United States [1, 2] and is responsible for cardiovascular diseases, type II diabetes, and certain cancers [3, 4]. Healthier eating behaviors such as increased fruit and vegetable consumption are associated with reduced risk of cardiovascular disease and certain cancers [5]. Interventions to increase healthier eating behaviors such as reducing saturated fat consumption have led to significant improvements in health outcomes such as reducing coronary heart disease [6, 7]. Mobile technology may enable the delivery of such dietary interventions in a cost-effective and scalable manner [8] given its increasing ubiquity [9], ability to reach individuals at nearly any time or place [10], and potential to deliver timely feedback, personalization, and interactivity to maximize intervention effectiveness [11]. An explosion of health-promoting mobile apps has occurred in recent years [12] including those to promote healthier eating [13–15]. However, most mobile apps are yet to incorporate an integrated set of theory-based strategies known to cause and sustain changes in health behaviors [16–18].

The process of developing such mobile health (mHealth) interventions is complex, requiring numerous decisions that integrate behavioral theory, user feedback, and technical and practical feasibility considerations. As a result, investigators have called for the thorough reporting of the mHealth intervention development process so that researchers may learn from each other’s experiences [19]. Moreover, the CONSORT guidelines recommend publishing a precise description of the final intervention [20], for which standardized language and descriptors may be useful [21]. While some researchers have notably published accounts of the iterative development and final versions of their mobile health interventions [13, 22–26], the relative lack of such reporting impedes advancements in mHealth research [22].

Several approaches to guide the development of more effective mHealth interventions have been proposed, including grounding interventions in behavioral theory [16–18]; incorporating brainstorming, rapid prototyping, and multiple stages of user feedback (i.e., user-centered design and design thinking) [27, 28]; and subjecting interventions to rigorous evaluation [29]. While numerous frameworks have been introduced to guide mHealth intervention development [19, 30–32], the IDEAS framework [manuscript under review] incorporates these approaches into a comprehensive, step-by-step process, and thus was used to guide the iterative development of Vegethon.

This research sought to do the following: a) develop an mHealth intervention to increase healthier eating behaviors among overweight adults; b) describe the iterative development of Vegethon, as guided by the IDEAS framework; and c) characterize the final intervention, including a detailed description of its components and theoretical grounding using standardized terminology specified by the taxonomy of behavior change techniques.

## Methods and results

**Fig. 1:**
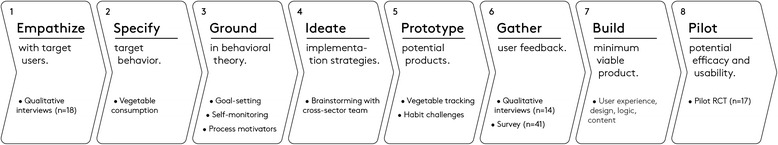
Application of the IDEAS framework to the development of Vegethon mobile app. *Empathize:* gather qualitative insights from users (e.g., in-depth interviews, focus groups). *Specify:* translate broad behavioural goals into a highly specific target behaviour, taking into consideration actionability, health impact, and user acceptability. *Ground:* ground intervention in behavioural theory and evidence and incorporate relevant behavioral strategies. *Ideate:* brainstorm creative strategies for translating theory and user insights into features, using inspiration from wide and varied sources. *Prototype:* develop rough prototypes of ideas rapidly and iteratively, sharing, discussing, and improving prototypes as a cross-sector team. *Gather:* gather user feedback on prototypes (e.g., interviews, questionnaires), and uncover insights to inform product improvement. *Build:* build a fully functional minimum viable product, and incorporate app analytics to collect data on app usage patterns. *Pilot:* conduct a small-scale evaluation of the intervention to test potential efficacy, feasibility, and usability, analyze usage behaviour, and refine study protocol

The intervention development process took the form of a collaborative academia-industry partnership in which researchers, product designers, engineers, and dietitians worked collaboratively throughout all phases. *IDEAS* [manuscript under review], a framework for developing digital behavioral health interventions, guided the process and was used for its integration of behavioral theory, user-centered design and design thinking, and evaluation. The first eight of ten IDEAS phases (Fig. 1) were undertaken: 1) empathize with target users (i.e., qualitative interviews); 2) specify target behavior; 3) ground in behavioral theory; 4) ideate implementation strategies; 5) prototype potential products; 6) gather user feedback (i.e., qualitative interviews; questionnaire); 7) build minimum viable product; and 8) pilot potential efficacy and usability (i.e., pilot RCT). As in other published accounts of mHealth intervention development, each phase was used to inform the next; therefore, the methods and results of each phase are presented sequentially [22].

### Phase 1. Empathize with users

To inform the development of the intervention, in-depth semi-structured qualitative interviews were conducted with an aim to better understand participants’ eating behaviors, mobile app usage, and challenges to eating more healthily. It has been asserted that participants have a limited understanding of what types of interventions would be most useful to them [33]. Thus, rather than set out to gather participants’ explicit ideas for mHealth interventions, this phase sought to equip our interdisciplinary team with a rich understanding of the beliefs, circumstances, and thought processes of our target population in order to facilitate the later ideation of more empathetic design solutions [34].

A target population of overweight adults enrolled in an ongoing 12-month weight loss study at Stanford University (parent trial) was selected due to participants’ motivation to eat more healthily and the practical feasibility of piggybacking onto a parent trial to assess the efficacy of an mHealth intervention using rigorously-collected parent trial data (e.g., BMI; 24-h dietary recalls). Participants were 18–50 years of age, had BMIs of 28–40 kg/m^2^, and lived in the geographical area surrounding Stanford University. As part of the parent trial, participants were randomly assigned to a low-fat or low-carbohydrate (carb) diet and encouraged to attend nutrition education classes every 1–4 weeks. Those interested in informing the design of an mHealth intervention were invited for interviews. In-depth interviews were conducted by one of the authors (SAM) and three research assistants. The interviews were audio-recorded and then transcribed verbatim. An applied thematic analysis strategy was applied to the interview transcripts by one of the authors (SAM) using NVivo, and findings were validated with all data collectors.

Eighteen adults (women/men: 9/9) aged 42.8 ± 6.9 years (mean ± SD) with body mass indices (BMIs) of 33.3 ± 4.0 kg/m^2^ were interviewed. Several themes emerged, including participants’ desire to be held accountable in a manner that would be more motivating, non-judgmental, and not overly time-consuming. When asked what type of mobile technology might help support healthier eating, participants almost always discussed an improved tool for self-monitoring. They cited self-monitoring as essential to eating more healthily but emphasized its time-consuming nature, their desire for it to be faster and more effortless, and their willingness to compromise on accuracy for simplicity.

*“The most important thing to stay on the rails is the tracking. But the goal for me, in tracking, is two minutes per day, not if it is more than 10 minutes…With tracking food, I don’t need to be very precise, and if I have to be very precise each time, it is a lot of pain and is not efficient.” (P7)*

Participants’ broader goals were to build a healthier lifestyle and lay the foundation for long-term health, wellbeing, and behavior change. Many reported the practice of planning meals in advance as central to following their diets given their busy lives. Participants who struggled to plan their meals noted the drawbacks: “I’m a modern person. I don’t plan for anything, and that is the problem.” These qualitative findings informed subsequent phases and indicated that possible intervention routes might include enabling simple and efficient self-monitoring of food intake, and supporting the rehearsal or formation of healthier meal planning routines.

### Phase 2. Specify target behavior

We next sought to select a specific target behavior that the intervention would aim to modify. A review of the existing evidence was conducted to identify potential target eating behaviors with significant health benefits. It was determined the target behavior would center around a whole food (e.g., vegetable) rather than nutrient (e.g., sugar) to increase the ease with which users could monitor their intake and therefore take action. This approach was consistent with recommendations to focus on whole foods rather than reductionist dietary patterns when seeking to modify dietary behavior [35, 36]. A review of the evidence for holistic dietary patterns suggested that the following behaviors offered significant health benefits: increased intake of fruits and vegetables [37–40]; reduced intake of sugar-sweetened beverages [41–43]; and reduced intake of animal products [44, 45]. Reduced intake of animal products was considered incompatible with the low-carb diet of the parent trial and was therefore discarded. Increased intake of fruits and vegetables was determined to be preferable to reduced intake of sugar-sweetened beverages due to its positive framing [46] and its potential to have a cascade effect [47], positively influencing other dietary behaviors by replacing the consumption of more caloric foods. To reinforce the potential cascade effect and weight loss goals of the parent trial, the target behavior was further specified to focus only on non-starchy vegetables, excluding fruits and starchy vegetables for their greater sugar and caloric content and comparatively weaker evidence base for a health benefit [48].

Phases 1 and 2 were conducted concurrently to confirm the selected target behavior with user feedback. Participants’ self-reported usual vegetable consumption ranged from 1 to 10 servings daily (1 serving = 1 fist size), and participants were either open to increasing their consumption or satisfied with their current consumption. One user noted, “I am happy with the amount [of vegetables I am eating],” while another said, “I wasn’t a huge vegetable eater before this study…and I would love to increase [the amount of vegetables I eat].” These findings confirmed the decision to select *increased consumption of non-starchy vegetables* as the target behavior.

### Phase 3. Ground in behavioral theory

Numerous theory-based strategies were identified for possible inclusion. Goal-setting [49] and self-monitoring [50] were considered due to their suitability for mobile technology [51] and alignment with user feedback. Habit formation was considered due to its demonstrated efficacy in changing dietary behavior [52, 53] and alignment with user insights. To enhance the intrinsic motivation of increasing vegetable consumption, process motivators [54] that focused on making the processes of self-monitoring and vegetable eating rewarding were considered, including elements of fun, challenge, choice, control, curiosity, context, and personalization [55]. Several other strategies were considered, including, but not limited to: reframing [56], social comparison [57], gamification [58], growth mindset [59], and identity revision [60].

### Phase 4. Ideate creative implementation strategies

With potential behavioral strategies identified, the team engaged in a series of group brainstorming sessions, also known as ideation [61], to conceive of creative ways in which these techniques might be implemented. Brainstorming centered around potential mobile app features and was inspired by a collection of highly rated apps on the App Store. A multidisciplinary team enabled the ideation of a wide range of diverse ideas taking behavioral theory, user insights, and product experience into account.

### Phase 5. Prototype potential products

From these ideas, potential versions of an app were sketched, shared, and discussed to identify the most promising prototypes. Benefits of creating early-stage prototypes include the ability to quickly and cheaply gather feedback on many different possible intervention approaches prior to investing significant resources in any one particular approach [62].

Two core prototypes emerged, *vegetable tracking* and *habit challenges*, which were developed into roughly-sketched, digital clickable prototypes with which participants could interact. The vegetable tracking prototype enabled simple and efficient self-monitoring of vegetable consumption, while the habit challenges prototype enabled the selection and rehearsal of new vegetable consumption habits. A constellation of secondary features (*goal setting*; *daily inspiration*; *leaderboard*; *progress*; *push notifications*) were also prototyped to further engage users and increase intervention potency.

### Phase 6. Gather user feedback

#### Qualitative interviews

To gather user feedback on the prototypes, qualitative interviews were conducted with an aim to determine which prototype users preferred and ways in which both prototypes could be improved. The same target population, methods for data collection and analysis, and human subjects approval were used as in phase 1. Fourteen adults (women/men: 11/3) aged 42.6 ± 8.4 years (mean ± SD) with body mass indices (BMIs) of 32.6 ± 3.2 kg/m^2^ were interviewed. Six key themes emerged including: the need for a focus on vegetable self-monitoring; the inclusion of vegetable challenges; the simplification of features; advice and inspiration for eating vegetables; reminder notifications; and peer comparison.

##### A focus on vegetable self-monitoring

Overall, participants expressed positive interest in the vegetable tracking prototype, stating that it was “simple,” “easy,” and “a great idea”. While some were concerned that it might be burdensome to use the app in addition to more holistic calorie-tracking apps such as MyFitnessPal, most participants expressed excitement about the intervention prototype.

*“This would be great because it is a very easy way to track [vegetables], and an official way to see progress…It is pretty much like making it easy, so you can’t be lazy.” (P8)*

When comparing the two core prototypes (vegetable tracking vs. habit challenges), participants favored the vegetable tracking prototype. They noted that the primary purpose of the app, in their view, would be tracking their vegetable consumption, and that completion of habit challenges would be “secondary”.

*“The fact that you are trying to change your habit and develop a daily habit in terms of having [vegetables] as snacks, or adding them to your breakfast, is a secondary thing. So to me, the important thing is the fact that you are having five servings of vegetables per day, or hitting the target that you have set yourself.” (P26)*

Participants also reported frequently consuming the same types of vegetables, and the desire to easily track the vegetables they more frequently consumed using a “list” or “filter” so that logging “would be more efficient.”

##### Inclusion of vegetable challenges

Despite an overall preference for the vegetable tracking prototype, some participants confirmed interest in the habit challenges prototype, expressing their inclination to try the challenges because they seemed “fun”.

*“I do like the idea of it being a challenge because it’s sort of competitive, and even with myself, to think, ‘I did it.’ I like that.” (P33)*

Users desired a greater number and variety of possible challenges to choose from, in addition to the four challenges presented in the initial prototype.

##### Simplification of features

Important practical complications were raised by participants with regard to the premise of selecting one daily habit challenge per week. The need for simplifying the challenges to avoid potential confusion emerged. Similarly, while participants were interested in the progress feature, there was confusion about the information displayed.

*“I guess the bar graph here doesn’t really make sense to me…because you are saying on here, “50 %, 3.5 servings, today”. 50 % of what? 50 % of my goal? 50 % of the vegetables?” (P24)*

##### Advice and inspiration for eating vegetables

Participants expressed their interest in the ‘advice’ functionality. They wanted to see the “benefits” of consuming more vegetables and be “inspired” to increase their consumption. They also reported wanting “ideas” with “specific” suggestions for incorporating vegetables into their meals and overcoming the “boredom” of eating the same meals repeatedly.

*“You know, if I am trying to get six servings a day, and I am only getting four, what are some ideas where I can incorporate them that I haven’t thought of…like precooking your asparagus the night before by steaming it as you prepare dinner. For me, or even others in my cohort, we would benefit from things that are basic, especially those of us who are not traditional vegetable eaters.” (P8)*

##### Reminder notifications

Users were also interested in receiving notification messages to “remind” them to log their vegetable intake or to notify them of their progress. They noted that these types of reminders served as “a good nudge.” However, participants varied notably in their desired frequency of such notifications. Some wanted to receive notifications several times throughout the day, while others wanted to receive no more than one notification every 4–5 days. Participants also expressed a desire to be able to adjust the frequency of notification timing to suit their personal schedules, lifestyles, and changing app usage preferences over time.

##### Peer comparison

Participants were interested in competing with others and “liked the way [the app] ranked everybody.” They believed they would be more motivated by competing against “peers,” such as the classmates from their parent trial health education classes. By contrast, many were not interested in competing against friends and family outside of the study. Participants cautioned that they might become discouraged if they were too far behind, thinking, “I’m already behind; why bother?” Some wanted the leaderboard to be anonymous while others did not; however, all agreed an anonymous leaderboard would hold them less accountable and “wouldn’t have as much impact.”

#### Questionnaire findings

To complement these qualitative findings, a questionnaire with 5-point Likert-type response categories was developed to assess interest in the features under consideration: *goal setting & progress*; *vegetable tracking*; *daily inspiration*; *challenges*; *leaderboard*; and *personalized notifications*. A *vegetable pictures* sharing feature was added in response to a user’s suggestion and overall feedback indicating a desire to be inspired. Forty-one adults (women/men: 23/18) aged 44.1 ± 6.4 years (mean ± SD) with body mass indices (BMIs) of 33.2 ± 3.2 kg/m^2^ completed the questionnaire. Participants were largely interested in all features. The only feature in which more were uninterested (i.e., responding “probably not” or “no”: 46 %) than interested (i.e., responding “absolutely” or likely”: 29 %) was *vegetable pictures*.

#### App refinements

Based on these findings, several decisions and adjustments were made. The prototype enabling vegetable self-monitoring was selected. A mechanism for marking “favorite” vegetable types was added to allow users to more quickly log the vegetables they most frequently consumed. The habit challenges, which participants had found to be useful but secondary to self-monitoring, were redesigned to support the primary self-monitoring feature. Rather than new habits requiring additional self-monitoring (e.g., packing snack baggies of carrots every morning), the new challenges focused on behaviors already reflected by self-monitoring (e.g., eat 2 servings of broccoli). This design adjustment enabled challenges to be fulfilled automatically via self-monitoring, requiring no additional effort by the user. *Progress* graphs were simplified and explained more explicitly to avoid confusion. *Daily inspiration* was renamed *advice and tips* and further developed. *Push notifications* were designed to nudge the user nightly but reduce in frequency if participants stopped using the app, to limit annoyance. The *leaderboard* was further developed to: compare users to other parent trial participants; display users’ first names to increase accountability; and match users according to their baseline vegetable consumption to reduce potential discouragement. As with all technologies, it was not possible or advantageous to include all features suggested by users. Omitted suggestions included the ability to adjust the timing of notifications and the ability to share *vegetable pictures*, a feature which was not supported by the questionnaire findings.

### Phase 7. Build minimum viable product

A single fully functional mobile app prototype was developed. This stage focused on a further level of design detail, including: a) user experience; b) visual design; c) logic (e.g., for graphs, notifications); and d) content. User experience and visual design were informed by product designers, logic by researchers and statisticians, and nutritional content by dieticians. Numerous small and large decisions were made to translate a rough prototype into a product suitable for mainstream delivery. Decisions were made together by the cross-sector team at every point to ensure that user experience and theoretical grounding were maximized throughout. For example, an image (i.e., a stack of vegetables) was selected for the splash page for both its visual appeal and its ability to help reframe the goal to consume a minimum number of vegetables as a positive and exciting endeavor rather than an unpleasant chore. In addition, the messages sent via notifications were written to be friendly and positive in tone while incorporating appropriate behavior change techniques, such as referring to users as ‘Vegethoners’ to help them begin to construct a new self-identify as vegetable eaters.

### Phase 8. Pilot test

A pilot RCT [63] was conducted to assess the potential efficacy, usability, and acceptability of the first complete version of the app. Parent trial participants were re-randomized to the use of *Vegethon* or a waiting-list control condition. Differences in vegetable consumption were assessed using an adapted Harvard Food Frequency Questionnaire (FFQ) at baseline and 12 weeks post-randomization. App usability and satisfaction were measured with a post-intervention questionnaire. Seventeen overweight or obese adults aged 42.0 ± 7.3 years (mean ± SD) with BMIs of 32.0 ± 3.5 kg/m^2^ were randomized. Consumption of overall vegetables, green leafy vegetables, dark and yellow vegetables, and cruciferous vegetables were significantly greater in the intervention versus control condition (*p* = 0.02) [63]. Participants reported positive experiences, including strongest agreement with the statements: “I have found Vegethon easy to use” and “I would recommend Vegethon to a friend.” App usage was measured automatically using inbuilt software; among the 8 participants randomized to the intervention, 5 used the app on a regular basis (i.e., not missing more than 5 consecutive days) for 3.7 ± 2.0 weeks (mean ± SD) (range: 0.9 to 6.0 weeks) during the 6-week intervention. Based on these findings, strategies were explored to increase the degree and duration of user engagement, and technical enhancements were made including increasing responsiveness speed and resolving engineering bugs.

### Final intervention and theoretical grounding

**Fig. 2:**
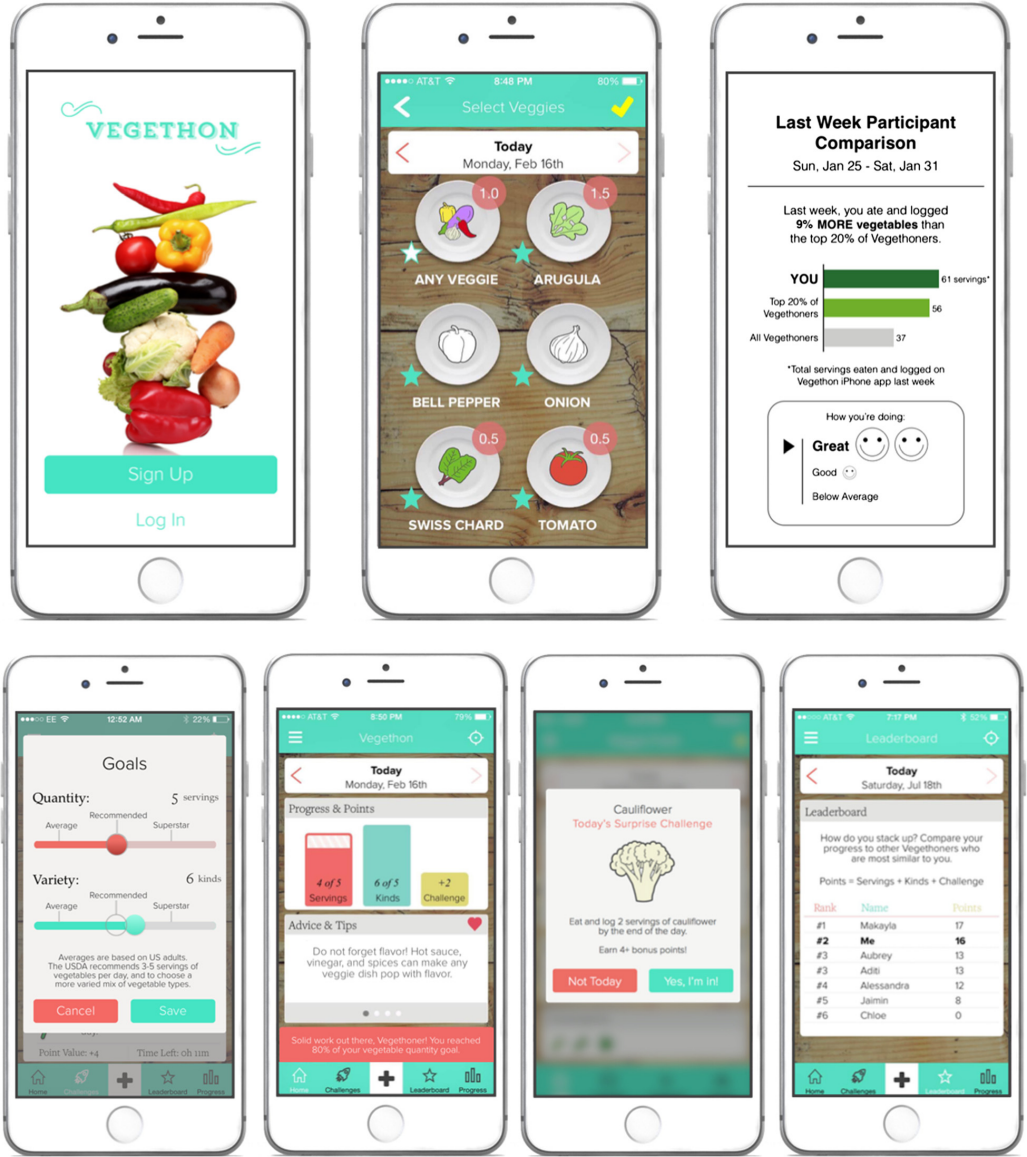
Vegethon features: splash, select veggies, weekly reports, goals, home, surprise challenges, and leaderboard. *Splash:* startup page framing the intervention as a challenge to eat as many vegetables as possible (13.2^a^ framing/reframing). *Select veggies:* self-monitoring of vegetable consumption by tapping on any of 30 icons representing individual vegetable types (2.3^a^ self-monitoring of behavior). *Weekly reports:* weekly social comparison of consumption to other Vegethoners (2.2^a^ feedback on behavior, 6.2^a^ social comparison, 6.3^a^ information about others’ approval). *Goals:* goal setting for daily number of vegetable servings and types (1.1^a^ goal setting (behavior)). *Home*: a) *progress & points:* feedback on today’s vegetable consumption vs. goals (1.6^a^ discrepancy between current behavior and goal, 2.2 feedback on behavior); b) *advice & tips:* nutritional and behavioral information (4.1^a^ instruction on how to perform a behavior); c) *in-app notifications:* feedback on fulfillment of goals and challenges (2.2^a^ feedback on behavior, 10.4^a^ social reward, 13.5^a^ identify associated with changed behavior). *Surprise challenges:* challenges to consume a specific vegetable type and quantity; delivered through in-app pop-up notifications every 1–4 days, with the choice to accept or decline (1.9^a^ commitment). *Leaderboard:* daily social comparison of progress to six similar peers, using a point-based system encompassing vegetable servings, vegetable types, and challenges (6.2^a^ social comparison). ^a^Behavior change technique (BCT), listed by Michie *et al.*‘s taxonomy number [21]

The final Vegethon mobile intervention [64], currently being evaluated in an RCT, is presented in Fig. 2 and is coded for its inclusion of behavior change techniques in Table 1. Overall, Vegethon is a stand-alone mobile app that enables vegetable consumption self-monitoring and focuses on making the *process* of behavior change rewarding [54] (e.g., the fun of receiving a surprise vegetable challenge; the pride in surpassing a peer’s vegetable score). This process motivation strategy stands in contrast to strategies focusing on the eventual outcomes of behavior change (e.g., avoidance of a heart attack) that are often too far in the future to motivate and sustain day-to-day behavior changes [54]. Interventions emphasizing the *process* of behavior change in this way may be more effective in initiating and sustaining behavioral changes due to their efficacy in cultivating intrinsic motivation [54, 55].

**Table 1:** Vegethon mobile app intervention mapped to behavior change techniques (BCTs)

Behavior Change Technique^a^	Definition^b^	Intervention component and description
1.1. Goal setting (behavior)	Set or agree on a goal defined in terms of the behavior to be achieved	Goals: select daily goals for number of servings and types of vegetables to consume
1.6. Discrepancy between current behavior and goal	Draw attention to discrepancies between current behavior and previously set behavioral goals	Progress & points; progress: bar graphs showing the discrepancy between goals and recorded consumption
1.9. Commitment	Ask the person to affirm or reaffirm statements indicating commitment to change the behavior	Surprise challenges: users are prompted to select “I’m in!” to affirm their commitment to undertake a surprise challenge
2.2. Feedback on behavior	Monitor and provide informative or evaluative feedback on performance of the behavior	In-app notifications: notifications when users reach 100 % of their daily goal or a 3-day or 7-day vegetable logging streak
2.3. Self-monitoring of behavior	Establish a method for the person to monitor and record their behavior	Select veggies: self-monitoring of vegetable servings and kinds consumed
4.1. Instruction on how to perform a behavior	Advise or agree on how to perform the behavior	Advice & tips: information on how to cook or prepare different vegetable types, and ideas for completing challenges
5.1. Information about health consequences	Provide information about health consequences of performing behavior	Advice & tips: information on health benefits of consuming vegetables
6.2. Social comparison	Draw attention to others’ performance to allow comparison with the person’s own performance	Leaderboard; weekly reports: comparing users’ consumption with others similar to them
6.3. Information about others’ approval	Provide information about what other people think about the behavior.	Weekly reports: great, good, or below average labels corresponding to participant performance
7.1. Prompts/cues	Introduce or define environmental or social stimulus to cue behavior	Push notifications: notifications to prompt self-monitoring of vegetable consumption
7.2. Reduce prompts/cues	Withdraw gradually prompts to perform the behavior	Push notifications: reduction in frequency if user has stopped logging, to reduce likelihood of annoyance
9.1. Credible source	Present verbal or visual communication from a credible source in favor of or against behavior	Goals: indicate the ‘recommended’ daily veg intake from the USDA
10.4 Social reward	Arrange verbal/non-verbal reward if and only if effort/progress is made	In-app notifications: messages to notify the user that a goal or challenge was met
10.5. Social incentive	Inform that verbal or non-verbal reward will be delivered if and only if effort/progress is made	Challenges: challenges with point-based rewards that will be delivered if met
13.1. Identification of self as role model	Inform that one’s own behavior may be an example to others	Push notifications: messages that label users as role models (e.g. You’re setting an impressive example in the Vegethon community.)
13.2. Framing/reframing	Suggest adoption of new perspective on a behavior to change cognitions/emotions about it	Name, tutorial: overall intervention framed as a race to eat as many vegetables as possible (rather than meeting a minimum necessary threshold)
13.5. Identity associated with changed behavior	Advise the person to construct a new self-identity	Push notifications; in-app notifications: messaging to help users begin to identify themselves as vegetable eaters (e.g., calling users ‘Vegethoners’)
15.1. Verbal persuasion about capability	Tell the person they can successfully perform the wanted behavior	Push notifications: positivity even when participants haven’t met goals or interacted with app recently

^a^Listed by Michie *et al.*‘s taxonomy number [21]

^b^Definition summarized based on Michie *et al.*‘s taxonomy [21]

#### App name and premise

To exploit the persuasive qualities of gamification [65], the app is framed as a competition to eat as many vegetables as possible and named *Vegethon* to elicit notions of sustained competition associated with a marathon. When registering for an app account, participants complete an in-app tutorial that frames the intervention using process motivators including challenge and taste [54] to enhance the intrinsic motivation of increasing vegetable consumption. Participants are called “Vegethoners” to foster a process of identity revision towards one who is a vegetable eater [60] and to create a sense of community and conformity around the challenge to eat more vegetables [66]. This approach aligns with user feedback to challenge users by inspiring them.

#### Goals

Users are initially prompted to set *goals* for the quantity (i.e., servings) and variety (i.e., types) of their vegetable consumption using two visual analog scales. A daily time frame is used to align with national recommendations [67] and enable more proximal goals shown to more highly motivate behavior change [68]. To increase perceived choice and control [55], participants are prompted to personalize their goals within a range of 1–10 and are able to adjust these at any time. Default values requiring no action, which are known to influence decision making [69], are set to 5 to align with USDA recommendations and labeled “recommended.” Anchoring, also known to influence decision making [70], is used to indicate: a) “2” as “average,” to encourage low vegetable consumers to set realistic goals that support self-efficacy in goal attainment; and b) “8” as “superstar” to encourage high consumers to set more ambitious goals, to maintain an appropriate level of challenge.

#### Select veggies

*Select veggies* is the central app feature and enables self-monitoring of vegetable consumption. To minimize the time and effort involved while maximizing opportunities for fun and visual appeal [54], a single tap on a vegetable icon (e.g., eggplant, carrots) causes it to light up and increase its recorded quantity in increments of ½ servings. Vegetable logging is easily accessible via a large white “+” icon located on every screen. Users are instructed to estimate one serving as approximately the size of their fist; this approach sacrifices a degree of accuracy for simplicity, to encourage sustained self-monitoring. Qualitative findings suggested users are interested in recording their food consumption as effortlessly and efficiently as possible, and that this approach is acceptable to them.

#### Challenges

To integrate elements of gamification [65], seven ongoing *challenges* reward users with points and in-app notifications upon fulfillment. These challenges represent varying degrees of difficulty and reinforce the primary goals of the intervention: increased vegetable consumption and increased frequency of self-monitoring (e.g., “Breakfast champ: eat any vegetable before 11 am”). In addition, *surprise challenges* are pushed to users every four days and designed to encourage consumption of new vegetable types. Each surprise challenge enables perceived choice and control [55] through the selection of a preferred vegetable type and the ability to accept (“I’m in!”) or decline (“Not today”) the resulting challenge.

#### Advice and tips

*Advice and tips* are displayed on the home page, to provide inspiration in line with user feedback. Tips are positive, encouraging, and theory-driven. Some focus on framing the process of eating vegetables as enjoyable and delicious [54] (e.g., “Spice it up! Hot sauce is full of flavor and no added salt or sugar. Red, green, spicy, or mild Sriracha can change your taste for veggies.”). Others focus on increasing one’s growth mindset [59] (i.e., belief that effort can lead to change) around becoming a vegetable lover (e.g., “Did you know? The more you eat vegetables, the more your brain rewires to develop a preference for their taste.”).

#### Home, progress, in-app notifications, and leaderboard

Several types of feedback (i.e., informative, evaluative, and comparative) are used. Informative feedback is provided via bar graphs on the *home* and *progress* screens, indicating the quantity and variety of vegetables consumed, using daily, weekly, and monthly timeframes. Evaluative feedback is provided via tailored congratulatory *in-app notifications* when challenges and goals are met; to maximize persuasion, this feedback states both the evaluation (e.g., “Congratulations!”) and evaluated behavior (e.g., “You completed the Variety King challenge”) [71]. Comparative feedback [57] is provided via a *leaderboard* which displays progress in comparison to six other participants similar in gender and baseline self-reported vegetable consumption.

#### Push notifications

Push notifications are employed as: a) just-in-time reminders; b) encouragement; and c) re-engagement tools. Positive and non-judgmental language is used to build self-efficacy and avoid adverse effects on mood that may cause discontinued app use [46]. To maximize notification relevance, each message aligns with recent self-monitoring behavior. To minimize annoyance and the interruption of daily activities, notifications are sent during lunchtime and evening hours no more than once per day. Nine o’clock p.m. is used as the usual reminder time, as it is late enough that the majority of food for the day has likely been consumed, but early enough that there is still enough time to log before bed. This approach aligns with findings suggesting behavior change can be facilitated via apps that generate positively framed alerts that are relevant and timely but not overly frequent [46].

#### Weekly report

To increase user engagement, a personalized weekly report, *Your Vegethon report*, informs participants how their vegetable consumption compares to: a) other Vegethoners; and b) the top 20 % of Vegethoners. This report is adapted from an effective intervention developed by Cialdini and colleagues to reduce household energy consumption [72] and uses descriptive norms, which have demonstrated effectiveness in reducing energy consumption [73, 74] and reducing alcohol consumption [75]. Injunctive norms (i.e., feedback regarding the desirability of one’s current performance) are incorporated to prevent the boomerang effect that normative messages sometimes have on those performing above average [72]: a “great,” “good,” or “below average” rating, with corresponding happy or sad faces, are given to encourage low-achievers to boost vegetable consumption and high-achievers to maintain rather than reduce consumption. Aligning descriptive and injunctive norms in this manner has been shown to produce the greatest behavioral changes [76].

## Discussion

### Overview

Vegethon [64] is a theory-driven, user-informed mobile intervention that was systematically developed using the IDEAS framework. Vegethon targets increased vegetable consumption among overweight adults who are trying to lose weight and was coded for its inclusion of 18 behavior change techniques (BCTs) as specified by the taxonomy of 93 BCTs [21]. Self-monitoring, which was desired by users and supported by theory, emerged as the core component of the mobile app and was complemented by a constellation of theory-driven features to increase user engagement and intervention potency. A focus on process motivation guided overall intervention development to increase intrinsic motivation for self-monitoring and increasing vegetable consumption. The development of Vegethon involved numerous decisions throughout the process to integrate theoretical grounding, user feedback, and feasibility constraints; the way in which we combined these perspectives may be useful to researchers in the development of their own mHealth interventions. Vegethon demonstrated efficacy in a pilot RCT [63] and is currently being evaluated in a larger-scale RCT.

### Strengths

Among the strengths of this work is a description of the complete intervention development process, including a detailed characterization of the final intervention using standardized terminology. It demonstrates the integration of behavioral theory, user-centered design, design thinking, and pilot evaluation in the development and refinement of an mHealth intervention. The approach we used is a departure from traditional methods in which a technology is initially designed by researchers and subsequently developed by third-party contractors; the use of a cross-sector team throughout the process comprised of both researchers and technical developers has been called for by others [27] and may present an improvement on prior approaches.

### Limitations

It has been proposed that traditional behavioral theories may be too static in nature to inform the use of the dynamic just-in-time capabilities of mobile technology [17] that allow interaction based on data gathered through sensing technology [11]. While the use of theory-driven behavioral strategies was sufficient to guide the development of Vegethon, future intervention iterations may benefit from the use of more complex intervention development processes [77]. In addition, the users who informed the development of Vegethon were concurrently enrolled in a weight loss trial, were an average of 43 years old, and were interested in helping with the development of mobile technology. It is therefore possible that the resulting intervention may not be as suitable to other samples that are not enrolled in a weight loss trial, are younger and potentially interested in different types of engagement with mobile technology, and/or are not as highly interested or motivated to use a mobile app to increase their vegetable consumption. Future studies may investigate whether behavior changes are observed among other populations and/or whether these changes are sustained once the intervention has ended.

## Conclusions

The development of Vegethon was guided by the IDEAS framework and a cross-sector team, and involved numerous stages pursued iteratively and in quick succession. The resulting mHealth intervention is theory-grounded, user-informed, and supported by user feedback and findings from a pilot trial. The final intervention aims to increase vegetable consumption and is currently undergoing larger-scale evaluation in a randomized controlled efficacy trial among overweight adults enrolled in a weight loss trial.

## Abbreviations

app, mobile application; BCT, behavior change techniques; FFQ, food frequency questionnaire; mHealth, mobile health; UI, user interface; USDA, United States Department of Agriculture
